# Supplementary material for: Demolition Activity and Elevated Blood Lead Levels among Children in Detroit, Michigan, 2014–2018
Source: Int J Environ Res Public Health. 2020 Aug 19;17(17):6018. doi: 10.3390/ijerph17176018 (PMC7503460; doi:10.3390/ijerph17176018)
Supplement: Supplementary file 1 [file ijerph-17-06018-s001.zip › ijerph-860824-supplementary-proof.pdf]

Supplementary Materials: Demolition Activity and Elevated Blood Lead Levels among Children in Detroit, Michigan, 2014–2018

Demolition data are available from Detroit's Open Data Portal: https://data.detroitmi.gov/datasets/completed-residential-demolitions. Individual blood lead data cannot be made public due to privacy concerns.

Table S1. Results of a case-control analysis of the association between demolitions within 400 feet and incident elevated blood lead levels among children <6 years old, by calendar year, stratified by target ZIP codes, Detroit, 2014–2018 (N = 54,150 observations, 5430 cases).

Target ZIP Codes Only[a]	**2018**	**2017**	**2016**	**2015**	**2014**	**Overall**
Incident EBLL cases	241	284	318	250	313	1,406
Controls	1,156	1,197	1,239	1,075	1,198	5,865
	OR	OR	OR	OR	OR	OR
	95% CI	95% CI	95% CI	95% CI	95% CI	95% CI
1 Demolition before test	1.5	1.29	0.37	1.59	1.05	1.12
	(0.68, 3.08)	(0.60, 2.57)	(0.11, 0.96)	(0.80, 3.06)	(0.47, 2.14)	(0.80, 1.54)
2 or More Demolitions before test	0.73	1.66	3.90	0.75	2.75	2.09
	(0.04, 4.12)	(0.63, 4.03)	(1.68, 9.13)	(0.16, 2.62)	(1.20, 6.23)	(1.36, 3.16)
1 Demolition after test	1.24	1.44	1.68	1.82	1.2	1.36
	(0.59, 2.41)	(0.75, 2.66)	(0.73, 3.59)	(0.93, 3.42)	(0.65, 2.12)	(1.02, 1.80)
2 or More Demolitions after test	2.43	2.11	1.43	0.57	3.82	2.01
	(0.74, 7.00)	(0.94, 4.46)	(0.47, 4.02)	(0.09, 2.23)	(1.67, 8.77)	(1.33, 3.01)
Outside Target ZIP Codes	**2018**	**2017**	**2016**	**2015**	**2014**	**Overall**
Incident EBLL cases	706	729	951	745	893	4,024
Controls	8,487	8,904	8,930	7,760	8,774	42,855
	OR	OR	OR	OR	OR	OR
	95% CI	95% CI	95% CI	95% CI	95% CI	95% CI
1 Demolition before test	0.86	1.57	1.42	1.24	1.02	1.21
	(0.57, 1.26)	(1.05, 2.27)	(1.05, 1.89)	(0.88, 1.71)	(0.71, 1.43)	(1.04, 1.41)
2 or More Demolitions before test	0.95	2.29	1.99	1.07	1.52	1.53
	(0.39, 1.96)	(1.14, 4.27)	(1.17, 3.27)	(0.59, 1.80)	(0.94, 2.36)	(1.19, 1.94)
1 Demolition after test	0.93	1.37	1.1	0.83	1.32	1.07
	(0.61, 1.36)	(0.91, 1.99)	(0.75, 1.56)	(0.55, 1.20)	(0.95, 1.80)	(0.91, 1.26)
2 or More Demolitions after test	0.75	0.99	0.7	0.94	0.92	0.85
	(0.33, 1.48)	(0.38, 2.19)	(0.31, 1.40)	(0.47, 1.72)	(0.50, 1.56)	(0.62, 1.13)

Note: Cases were defined as the first venous test with a result ≥5 μg/dL; for individuals who did not have any venous tests during 2012-2018, the first test from a capillary or unknown specimen type with a result ≥5 μg/dL was considered an incident EBLL. The date the specimen was collected was used as the incident test date. Controls were individuals without an EBLL during the study period, the incident test date was the first result from a venous where available and from a capillary test where no venous was available. Results were estimated in logistic regression models adjusted for specimen type (venous, capillary, unknown), month and year of collection, age category (<1, 1-2, 3-5 years), gender, and ZIP code. [a]Target ZIP codes are the 5 ZIP codes with the highest percentage EBLL among children tested in 2016: 48202, 48204, 48206, 48213, 48214.

Table S2. Results of a case-control analysis of the association between demolitions within 400 feet and incident elevated blood lead levels among children <6 years old, by calendar year, varying exposure window in time, Detroit, 2014–2018 (N = 54,150 observations, 5430 cases).

	**2018**	**2017**	**2016**	**2015**	**2014**	**Overall**
Incident EBLL cases	947	1,013	1,269	995	1,206	5,430
Controls	9,643	10,101	10,169	8,835	9,972	48,720
15 Day Exposure Window						
1 Demolition before test	0.91	2.12	1.38	1.29	1.19	1.33
	(0.49, 1.56)	(1.28, 3.39)	(0.86, 2.15)	(0.82, 1.96)	(0.76, 1.81)	(1.08, 1.63)
2 or More Demolitions before test	0.58	3.59	3.43	1.36	1.35	1.84
	(0.09, 1.97)	(1.50, 8.00)	(1.59, 7.16)	(0.54, 2.91)	(0.66, 2.57)	(1.28, 2.59)
1 Demolition after test	0.87	0.99	1.01	0.96	1.26	1
	(0.47, 1.47)	(0.51, 1.76)	(0.57, 1.66)	(0.56, 1.55)	(0.78, 1.96)	(0.79, 1.26)
2 or More Demolitions after test	1.39	0.96	0.74	1.03	1.32	1.11
	(0.52, 3.12)	(0.32, 2.36)	(0.20, 2.12)	(0.35, 2.45)	(0.65, 2.47)	(0.74, 1.61)
30 Day Exposure Window						
1 Demolition before test	0.78	1.66	1.25	1.2	1.05	1.17
	(0.49, 1.19)	(1.11, 2.41)	(0.88, 1.74)	(0.83, 1.69)	(0.74, 1.48)	(0.99, 1.37)
2 or More Demolitions before test	0.66	2.10	2.97	0.87	1.59	1.63
	(0.20, 1.63)	(1.05, 3.93)	(1.75, 4.94)	(0.42, 1.63)	(0.98, 2.51)	(1.25, 2.09)
1 Demolition after test	1.13	1.41	0.87	1.1	1.43	1.15
	(0.76, 1.64)	(0.92, 2.08)	(0.57, 1.28)	(0.76, 1.56)	(1.02, 1.97)	(0.97, 1.35)
2 or More Demolitions after test	1.11	1.48	0.68	1.06	1.56	1.18
	(0.53, 2.08)	(0.75, 2.71)	(0.28, 1.43)	(0.51, 2.01)	(0.94, 2.49)	(0.89, 1.54)
60 Day Exposure Window						
1 Demolition before test	1.13	1.54	1.14	1.25	1.08	1.20
	(0.83, 1.51)	(1.12, 2.08)	(0.87, 1.47)	(0.95, 1.63)	(0.81, 1.43)	(1.06, 1.36)
2 or More Demolitions before test	1.09	2.37	1.88	1.00	1.70	1.58
	(0.59, 1.86)	(1.45, 3.74)	(1.29, 2.69)	(0.62, 1.56)	(1.19, 2.40)	(1.31, 1.90)
1 Demolition after test	1.06	1.28	1.18	0.96	1.07	1.09
	(0.79, 1.42)	(0.95, 1.70)	(0.87, 1.57)	(0.70, 1.28)	(0.82, 1.40)	(0.96, 1.24)
2 or More Demolitions after test	0.92	1.42	1.38	0.78	1.51	1.20
	(0.52, 1.52)	(0.84, 2.29)	(0.88, 2.12)	(0.44, 1.30)	(1.05, 2.12)	(0.99, 1.46)

Note: Cases were defined as the first venous test with a result ≥5 μg/dL; for individuals who did not have any venous tests during 2012-2018, the first test from a capillary or unknown specimen type with a result ≥5 μg/dL was considered an incident EBLL. The date the specimen was collected was used as the incident test date. Controls were individuals without an EBLL during the study period, the incident test date was the first result from a venous where available and from a capillary test where no venous was available. Results were estimated in logistic regression models adjusted for specimen type (venous, capillary, unknown), month and year of collection, age category (<1, 1-2, 3-5 years), gender, and ZIP code.

Table S3. Results of a case-control analysis of the association between demolitions within 200 foot circular buffer 45 days prior to a test and incident elevated blood lead levels among children < 6 years old, by calendar year, Detroit, 2014–2018 (N=54,150 observations, 5,430 cases).

	2018	2017	2016	2015	2014	Overall
Incident EBLL cases	947	1,013	1,269	995	1,206	5,430
Controls	9,643	10,101	10,169	8,835	9,972	48,720
Main analysis (includes demolitions 45 days pre- and post-test)	OR 95% CI	OR 95% CI	OR 95% CI	OR 95% CI	OR 95% CI	OR 95% CI
1 Demolition before test	0.75	0.86	1.72	1.22	1.53	1.29
	(0.38, 1.35)	(0.38, 1.69)	(1.09, 2.62)	(0.72, 1.97)	(0.99, 2.30)	(1.03, 1.60)
2 or More Demolitions before test	0.61	2.61	2.51	0.62	1.73	1.65
	(0.03, 3.00)	(0.69, 8.36)	(1.05, 5.58)	(0.10, 2.13)	(0.71, 3.78)	(1.02, 2.56)
1 Demolition after test	1.02	1	0.89	1.46	1.63	1.21
	(0.58, 1.69)	(0.52, 1.75)	(0.50, 1.49)	(0.91, 2.26)	(1.05, 2.45)	(0.97, 1.50)
2 or More Demolitions after test	1.31	3.04	1.2	1.94	1.16	1.59
	(0.30, 3.97)	(1.11, 7.62)	(0.37, 3.35)	(0.61, 5.17)	(0.46, 2.55)	(1.01, 2.43)

Note: Cases were defined as the first venous test with a result ≥5 μg/dL; for individuals who did not have any venous tests during 2012-2018, the first test from a capillary or unknown specimen type with a result ≥5 μg/dL was considered an incident EBLL. The date the specimen was collected was used as the incident test date. Controls were individuals without an EBLL during the study period, the incident test date was the first result from a venous where available and from a capillary test where no venous was available. Results were estimated in logistic regression models adjusted for specimen type (venous, capillary, unknown), month and year of collection, age category (<1, 1-2, 3-5 years), gender, and ZIP code.

Table S4. Results of a case-control analysis of the association between demolitions within 400 feet 45 days prior to a test and incident venous elevated blood lead levels among children < 6 years old, by calendar year, Detroit, 2014–2018 (N=42,311, 4,300 cases).

	**2018**	**2017**	**2016**	**2015**	**2014**	**Overall**
Incident EBLL cases	715	776	912	852	1,045	4,300
Controls	6,882	7,575	7,848	7,181	8,525	38,011
Main analysis	OR	OR	OR	OR	OR	OR
(includes demolitions 45 days before and after test)	95% CI	95% CI	95% CI	95% CI	95% CI	95% CI
1 Demolition before test	1.16	1.3	1.04	1.36	1.04	1.02
	(0.76, 1.73)	(0.82, 2.01)	(0.72, 1.49)	(0.96, 1.91)	(0.71, 1.48)	(0.62, 1.63)
2 or More Demolitions before test	0.68	2.02	3.02	1.24	1.89	0.75
	(0.19, 1.88)	(1.03, 3.88)	(1.76, 5.17)	(0.66, 2.22)	(1.22, 2.89)	(0.21, 2.18)
1 Demolition after test	1.04	1.03	1.02	0.95	0.99	1.04
	(0.65, 1.71)	(0.70, 1.55)	(0.67, 1.59)	(0.61, 1.53)	(0.66, 1.52)	(0.57, 1.97)
2 or More Demolitions after test	1.02	1.03	1.05	0.95	0.94	1.06
	(0.63, 1.70)	(0.69, 1.56)	(0.68, 1.66)	(0.60, 1.54)	(0.62, 1.44)	(0.58, 2.05)

Note: Cases were defined as the first venous test with a result ≥5 μg/dL; for individuals who did not have any venous tests during 2012-2018, the first test from a capillary or unknown specimen type with a result ≥5 μg/dL was considered an incident EBLL. The date the specimen was collected was used as the incident test date. Controls were individuals without an EBLL during the study period, the incident test date was the first result from a venous where available and from a capillary test where no venous was available. Results were estimated in logistic regression models adjusted for specimen type (venous, capillary, unknown), month and year of collection, age category (<1, 1-2, 3-5 years), gender, and ZIP code.

Table S5. Results of a cross-sectional analysis of the association between demolitions and prevalent elevated blood lead levels among all children < 6 years old tested for lead, Detroit, 2014–2018 (N=109,982, 8,658 cases).

	**2018**	**2017**	**2016**	**2015**	**2014**
EBLL cases (BLL ≥5 μg/dL)	1,406	1,658	2,055	1,624	1,915
BLL < 5 μg/dL	18,414	20,558	21,353	19,780	21,219
Main analysis	OR	OR	OR	OR	OR
(includes demolitions 45 days before and after test)	95% CI	95% CI	95% CI	95% CI	95% CI
1 Demolition before test	0.84	1.49	1.19	1.27	1.19
	(0.61, 1.13)	(1.15, 1.92)	(0.95, 1.46)	(1.01, 1.59)	(0.94, 1.49)
2 or More Demolitions before test	1.25	2.23	1.74	0.86	1.62
	(0.71, 2.04)	(1.43, 3.37)	(1.24, 2.39)	(0.56, 1.27)	(1.19, 2.17)
1 Demolition after test	0.95	1.29	1.29	1.11	1.2
	(0.71, 1.26)	(1.01, 1.64)	(1.03, 1.61)	(0.87, 1.41)	(0.96, 1.49)
2 or More Demolitions after test	1.17	1.12	0.96	1.23	1.35
	(0.71, 1.82)	(0.70, 1.72)	(0.61, 1.44)	(0.81, 1.81)	(0.98, 1.83)
Demolitions before test only	OR	OR	OR	OR	OR
	95% CI	95% CI	95% CI	95% CI	95% CI
1 Demolition	0.84	1.5	1.2	1.28	1.22
	(0.61, 1.13)	(1.16, 1.93)	(0.96, 1.48)	(1.02, 1.60)	(0.96, 1.53)
2 or More Demolitions	1.25	2.32	1.76	0.88	1.73
	(0.71, 2.04)	(1.49, 3.50)	(1.26, 2.43)	(0.57, 1.29)	(1.28, 2.30)

Note: Elevated Blood Lead Level (EBLL). Data from the Michigan Department of Health and Human Services Data Warehouse. Table includes one observation per child per year. Children can be included in multiple years. If a child had multiple tests in a year, only the highest result from a venous sample was retained. If no result from a venous sample was available, the highest result from a capillary or unknown sample was used. All models adjusted for age (<1, 1-2, 3-5 years), gender, ZIP code, month of test, and specimen type.

Table S6. Results of a case-control analysis of the association between demolitions and incident elevated blood lead levels among children < 6 years old, by calendar year, Detroit, 2014–2018 (N=54,150 observations, 5,430 cases).

	**2018**	**2017**	**2016**	**2015**	**2014**	**Overall**
Incident EBLL cases	947	1,013	1,269	995	1,206	5,430
Controls	9,643	10,101	10,169	8,835	9,972	4,8720
Main analysis<br>(includes demolitions 45 days before and after test)	OR<br>95% CI	OR<br>95% CI	OR<br>95% CI	OR<br>95% CI	OR<br>95% CI	OR<br>95% CI
≥ 1 Demolition before test (main exposure)	0.95<br>(0.69, 1.30)	1.66<br>(1.23, 2.20)	1.47<br>(1.15, 1.86)	1.24<br>(0.95, 1.60)	1.25<br>(0.97, 1.59)	1.30<br>(1.15, 1.46)
≥ 1 Demolition after test (negative control exposure)	0.97<br>(0.71, 1.30)	1.40<br>(1.05, 1.86)	1.09<br>(0.81, 1.45)	0.97<br>(0.72, 1.28)	1.35<br>(1.05, 1.71)	1.14<br>(1.01, 1.29)
Demolitions before test only	OR<br>95% CI	OR<br>95% CI	OR<br>95% CI	OR<br>95% CI	OR<br>95% CI	OR<br>95% CI
≥ 1 Demolition	0.95<br>(0.69, 1.29)	1.69<br>(1.26, 2.24)	1.47<br>(1.16, 1.87)	1.24<br>(0.95, 1.60)	1.30<br>(1.01, 1.65)	1.31<br>(1.17, 1.47)

Note: Main exposure and negative control exposure demolitions were dichotomized to 0, ≥1 demolitions within 45 days before and after test, respectively. Cases were defined as the first venous test with a result ≥5 μg/dL; for individuals who did not have any venous tests during 2012-2018, the first test from a capillary or unknown specimen type with a result ≥5 μg/dL was considered an incident EBLL. The date the specimen was collected was used as the incident test date. Controls were individuals without an EBLL during the study period, the incident test date was the first result from a venous where available and from a capillary test where no venous was available. Results were estimated in logistic regression models adjusted for specimen type (venous, capillary, unknown), month and year of collection, age category (<1, 1-2, 3-5 years), gender, and ZIP code

Table S7. Number of demolitions by census tract median year of housing construction, Detroit, 2014–2018.

	2014	2015	2016	2017	2018	2014-2018
Median Year Housing Built	n (%)	n (%)	n (%)	n (%)	n (%)	n (%)
1940s	1,285 (36%)	1,661 (41%)	1,234 (39%)	1,126 (45%)	1,500 (47%)	6,806 (41%)
1950s	1,069 (30%)	1,103 (28%)	734 (23%)	525 (21%)	912 (29%)	4,343 (26%)
1960s	37 (1%)	20 (1%)	20 (1%)	29 (1%)	26 (1%)	132 (1%)
1970s	12(<1%)	15 (<1%)	16 (1%)	4 (<1%)	11 (<1%)	58 (<1%)
1980s or later	74 (2%)	34 (1%)	44 (1%)	58 (2%)	32 (1%)	242 (1%)
Unknown	1,090 (31%)	1,182 (29%)	1,153 (36%)	766 (31%)	712 (22%)	4,903 (30%)
Total	3,567	4,015	3,201	2,508	3,193	16,484

Note: Census tract median year housing built data from American Community Survey, 2013-2017, Demolition data from City of Detroit Open Data Portal. Percentages may not sum to 100 due to rounding.
